# Glycemic control and vaccine response: the role of mucosal immunity after vaccination in diabetic patients

**DOI:** 10.3389/fimmu.2025.1577523

**Published:** 2025-05-08

**Authors:** Moaz A. Mojaddidi, Moutasem Aboonq, Saeed A. Alqahtani

**Affiliations:** Department of Basic Sciences, Taibah University, Medina, Saudi Arabia

**Keywords:** glycemic control, mucosal immunity, vaccine response, diabetes mellitus, immune cells

## Abstract

This review explores the critical interplay between glycemic control, mucosal immunity, and vaccine response in diabetic patients. Diabetes mellitus, characterized by impaired glucose regulation, significantly impacts immune function, particularly at mucosal surfaces. Poor glycemic control diminishes vaccine-induced antibody responses and compromises mucosal defenses, such as secretory IgA production, increasing susceptibility to infections. We synthesize evidence highlighting the importance of optimizing glycemic management prior to vaccination to enhance immunogenicity. Furthermore, we examine the potential of personalized vaccination strategies, tailored to individual glycemic status, age, BMI, and kidney function, to improve vaccine efficacy in this vulnerable population. Additionally, we discuss the role of adjunct therapies, including probiotics, nutritional interventions, and lifestyle modifications, in modulating the gut microbiota and reinforcing mucosal barrier integrity. This review underscores the necessity for an interdisciplinary approach, integrating metabolic management with innovative vaccine designs, to maximize protection against infectious diseases in diabetic patients. Future research should prioritize longitudinal studies assessing both systemic and mucosal immunity and refine personalized vaccination strategies to ensure robust and durable protection.

## Introduction

Diabetes mellitus, encompassing both type 1 and type 2 diabetes, presents distinct challenges in glycemic control due to differing pathophysiologies. Type 1 diabetes is characterized by an autoimmune destruction of pancreatic beta cells, leading to an absolute deficiency in insulin secretion, often manifesting acutely with symptoms such as polydipsia, polyphagia, and weight loss (1). In contrast, type 2 diabetes results from a combination of insulin resistance and a relative insulin secretory defect, typically progressing more gradually and often associated with obesity (1). Effective management of both types involves a multifaceted approach, including diabetes self-management education, self-monitoring of blood glucose, and medication adherence, which are crucial for preventing complications and improving quality of life (2). Studies indicate that children with type 1 diabetes generally possess satisfactory knowledge about glycemic control, although continuous education is recommended to maintain this awareness (3). Factors such as socioeconomic status, dietary knowledge, and self-efficacy significantly influence glycemic control, highlighting the need for personalized management plans that incorporate lifestyle modifications and psychological support (4). Technological advancements, such as continuous glucose monitoring (CGM) and digital health tools, further enhance diabetes management by providing real-time data and facilitating remote consultations (2). Overall, an integrated approach combining education, lifestyle changes, and technological innovations is essential for achieving optimal glycemic control in both type 1 and type 2 diabetes (2, 4).

Mucosal immunity plays a crucial role in the immune response to vaccines, particularly because many pathogens enter the host through mucosal surfaces, such as the respiratory and gastrointestinal tracts. Mucosal vaccines aim to induce protective immune responses at these entry points, offering a first line of defense by generating localized immune responses, including the production of secretory IgA and the activation of tissue-resident memory T cells (5, 6). Unlike traditional parenteral vaccines, which primarily elicit systemic immunity, mucosal vaccines can provide superior protection by targeting the site of pathogen entry, thus preventing infection and transmission more effectively (7, 8). The development of mucosal vaccines involves selecting appropriate antigens, delivery routes, and adjuvants to enhance their efficacy, as the unique anatomical and functional characteristics of the mucosal immune system require specialized approaches (9). Despite the challenges in developing effective mucosal vaccines, such as overcoming the mucosal barrier and ensuring stability against degradation, they offer significant advantages, including ease of administration, cost-effectiveness, and non-invasiveness, making them particularly suitable for use in resource-limited settings (7). Recent advancements in mucosal vaccine strategies, including novel adjuvants and delivery systems, have been pivotal during the COVID-19 pandemic, highlighting their potential in providing a dual-layered defense against respiratory pathogens (5). Overall, mucosal immunity is integral to the development of vaccines that can effectively prevent diseases at their point of entry, offering a promising alternative to conventional vaccination methods.

Glycemic control appears to be a critical factor in the mucosal immune response to vaccines in individuals with diabetes, as evidenced by several studies examining the relationship between glucose management and vaccine efficacy. Research indicates that poor glycemic control can impair the immune response following vaccination, as seen in patients with diabetes who exhibit lower antibody responses compared to non-diabetic individuals after receiving COVID-19 vaccines (10–12). Specifically, a study on type 1 diabetes patients demonstrated that pre-vaccination glucose control, particularly the time in range (TIR) and time above range (TAR), significantly correlates with stronger antibody responses post-vaccination, independent of HbA1c levels (13). This suggests that maintaining optimal glucose levels before vaccination can enhance immunogenicity. Furthermore, transient increases in blood glucose levels and insulin resistance have been observed following COVID-19 booster vaccinations in type 1 diabetic patients, highlighting the complex interplay between vaccination and glycemic control (14). Systematic reviews also underscore the importance of glycemic management in maximizing vaccine-induced immunogenicity, particularly with inactivated virus vaccines, although more extensive studies are needed to fully understand these dynamics (12). Overall, these findings suggest that effective glycemic control is essential for optimizing vaccine efficacy in diabetic patients, supporting the hypothesis that it plays a critical role in the mucosal immune response to vaccines.

The aim of this review is to explore the intricate relationship between glycemic control, mucosal immunity, and vaccine response in individuals with diabetes. By examining how fluctuating blood glucose levels impact mucosal immune responses post-vaccination, this review seeks to highlight the challenges faced by diabetics in achieving optimal vaccine efficacy. Additionally, we aim to investigate potential strategies, including glycemic management and novel vaccine formulations, that could enhance mucosal immunity and improve vaccine outcomes in this vulnerable population. Through this exploration, we hope to provide insights into optimizing vaccination strategies tailored to the unique immunological needs of diabetics.

## Glycemic control and immune function

Poor glycemic control significantly impacts immune cells, particularly macrophages and T cells, as well as mucosal immunity. Elevated glucose levels can impair the immune system by promoting excessive production of pro-inflammatory cytokines, which can lead to immune dysfunction and pathological conditions (15). In the context of diabetes, hyperglycemia is associated with chronic low-grade inflammation and immune dysregulation, which can exacerbate disease progression and complications (16). Specifically, high blood glucose levels in diabetic patients can reduce the count and function of innate immune cells, such as macrophages, and delay antigen presentation, thereby impairing the clearance of pathogens like *Mycobacterium tuberculosis* (17). This immune suppression is further evidenced by the blunted immune responses observed in diabetic patients with poor glycemic control, which is associated with a lower capacity for virus-neutralizing antibodies and a diminished CD4+ T cell response, increasing the risk of infections such as SARS-CoV-2 (18). Moreover, hyperglycemia promotes oxidative stress and the production of reactive oxygen species (ROS) in immune cells, which further exacerbates immune dysfunction by damaging cell structures and impairing immune cell signaling pathways. These changes can lead to a chronic pro-inflammatory state, making it difficult for the immune system to mount an adequate response to pathogens (19). Specifically, elevated glucose can induce the activation of the NF-κB pathway, which is known to increase the secretion of pro-inflammatory cytokines such as TNF-α and IL-6, further compromising immune responses. Further, chronic inflammation in both type 1 and type 2 diabetes exacerbates immune dysfunction, with type 1 diabetes linked to autoimmune responses and type 2 diabetes associated with systemic inflammation from insulin resistance. Additionally, poor glycemic control does not significantly alter the levels of salivary immunologic proteins, suggesting that while systemic immune responses are affected, mucosal immunity might remain relatively stable, although this does not preclude the risk of oral infections in diabetic patients (20). Additionally, glucose metabolism is crucial for T cell function, and dysregulation can lead to hyperactive immune responses or immune pathology, highlighting the need for tight regulation of glucose uptake to maintain immune homeostasis (19). The interplay between glucose levels and immune cell function is complex, as both hyperglycemia and hypoglycemia can alter immune mediators associated with macrophage and T cell activation, further complicating immune responses in diabetic conditions (21).

Resident and memory T cells are crucial for effective immune responses, particularly in the context of vaccines. Memory T cells, including tissue-resident memory T cells (TRM), provide long-lasting immunity by “remembering” previous infections or vaccinations and responding quickly upon re-exposure. These cells are particularly important in mucosal immunity, where they reside at entry points such as the respiratory and gastrointestinal tracts. In diabetic individuals, particularly those with poor glycemic control, the function of both resident and memory T cells is compromised. Chronic hyperglycemia has been shown to impair the activation, differentiation, and persistence of memory T cells, leading to a weakened immune response following vaccination. Furthermore, elevated blood glucose levels can reduce the ability of these cells to migrate to infection sites and mount a strong, localized immune response, which is critical for the effectiveness of mucosal vaccines.

In individuals with type 1 diabetes, impaired immune responses are primarily due to autoimmunity, while in type 2 diabetes, chronic inflammation related to insulin resistance plays a key role in the immune suppression observed. This divergence in immune dysregulation between type 1 and type 2 diabetes underscores the importance of personalized vaccination strategies. Chronic hyperglycemia can also impair T cell differentiation and reduce the efficacy of memory T cell responses, which are vital for long-term immunity, further diminishing the capacity of diabetic individuals to respond effectively to infections and vaccinations.

Overall, maintaining optimal glycemic control is crucial for preserving immune function and preventing complications in diabetic patients. The dysregulation of immune responses due to altered glucose metabolism underscores the importance of managing blood sugar levels to ensure that both innate and adaptive immune functions remain effective.

## Mucosal immunity and vaccine response

Mucosal immunity, particularly the role of secretory IgA, is crucial in the body’s defense against pathogens, especially those entering through mucosal surfaces such as the respiratory and gastrointestinal tracts. Secretory IgA is the predominant antibody isotype at these sites and is essential for neutralizing pathogens and preventing their adherence to epithelial cells, thus playing a pivotal role in mucosal vaccine responses (22, 23). The mucosal immune system is a complex network involving innate and adaptive components, including mucosal B and T cells, dendritic cells, and epithelial cells, which coordinate to produce IgA and other immune responses (23, 24). Mucosal vaccines, which aim to induce both systemic and mucosal immunity, are particularly promising as they can elicit strong local immune responses at the site of pathogen entry, offering a first line of defense (25–27). However, the development of effective mucosal vaccines is challenging due to the need for appropriate antigen delivery systems that can withstand the harsh mucosal environment and effectively target mucosa-associated lymphoid tissues (27). A key issue is the development of adjuvants that can enhance the immune response without causing excessive inflammation or damage to the mucosal tissues (28).

In healthy individuals, the mucosal immune system is adept at distinguishing between pathogenic and non-pathogenic antigens, maintaining tolerance to innocuous substances while mounting robust responses to harmful pathogens (28). However, in individuals with diabetes, mucosal immune responses can be altered. Diabetes is associated with immune dysregulation, which may affect the production and function of secretory IgA and other immune components, potentially leading to impaired vaccine responses (28). This altered immune landscape in diabetic individuals necessitates tailored vaccine strategies to ensure effective mucosal immunity. The development of mucosal vaccines that can induce both local and systemic immunity is crucial, particularly for populations with compromised immune responses, such as those with diabetes (29). Overall, while mucosal vaccines hold great promise, their efficacy can vary significantly between healthy individuals and those with underlying conditions like diabetes, highlighting the need for continued research and development in this field (22, 25, 28).

## Diabetes and impaired mucosal immunity

Elevated blood glucose levels, commonly associated with conditions such as obesity and diabetes, have a significant impact on mucosal immunity, particularly through the reduction of IgA production and the dysfunction of the epithelial barrier. Hyperglycemia has been shown to drive intestinal barrier permeability by altering the transcriptional programming of intestinal epithelial cells, which affects the integrity of tight and adherence junctions. This disruption facilitates the systemic influx of microbial products, thereby increasing the risk of enteric infections and systemic inflammation (30). The dysfunction of the mucosal immune system, particularly the mucosa-associated lymphoid tissue responsible for secretory IgA production, is also influenced by dietary changes and hyperglycemic conditions, which can lead to a compromised mucosal barrier and increased susceptibility to infections (31). Furthermore, hyperglycemia enhances the formation of neutrophil extracellular traps (NETs) in the oral mucosa, contributing to barrier disruption and inflammation, which underscores the broader implications of hyperglycemia on mucosal immunopathology (32). *In vitro* studies have demonstrated that high glucose exposure results in morphological and functional changes in the intestinal barrier, including reduced expression of junction proteins and increased permeability, which further compromises barrier integrity (33). These changes indicate broader dysregulation in trained immunity, where chronic basal inflammation impairs long-term immune responses to both infections and vaccines.

Trained immunity refers to the ability of the innate immune system to “remember” previous exposures to pathogens or vaccines, leading to a heightened response upon subsequent encounters. In diabetic individuals, chronic low-grade inflammation induced by hyperglycemia can alter this process, reducing the effectiveness of immune responses and vaccines. This inflammation can impact cytokine production, immune cell activation, and the ability of the immune system to “remember” pathogens, ultimately compromising both vaccine efficacy and infection defense.

Although hyperglycemia alone may not significantly alter intestinal permeability, it can potentiate inflammatory responses, such as the secretion of cytokines like IL-8, which exacerbate tissue inflammation and barrier dysfunction (34). A critical value in understanding this relationship is the maintenance of gut barrier integrity, which is essential for preventing systemic inflammation and autoimmune responses (35). Collectively, these findings highlight the critical role of hyperglycemia in impairing mucosal immunity by disrupting epithelial barrier function and reducing IgA production, thereby increasing vulnerability to infections and inflammation. Further, diabetes significantly impacts mucosal immunity, primarily through alterations in cytokine production and immune cell activity at mucosal surfaces. In type 1 diabetes, there is a notable reduction in key cytokines such as IL-17A, IL-22, and IL-23A within the gut mucosa, which is linked to inflammation rather than hyperglycemia. This cytokine imbalance contributes to impaired gut integrity and dysbiosis, characterized by a loss of segmented filamentous bacteria, which are crucial for maintaining mucosal immunity (36). Similarly, in type 2 diabetes, patients exhibit elevated levels of pro-inflammatory cytokines like IL-1, TNF, IFN, and IL-17A in the context of periodontal disease, indicating a heightened inflammatory response that exacerbates mucosal tissue damage (37). The loss of gut barrier integrity in type 1 diabetics can activate islet-reactive T cells, further linking mucosal immune dysfunction to autoimmune processes (38). Moreover, diabetes-induced immune dysfunction is characterized by impaired proliferation and senescence of immune cells, which parallels the concept of “inflammaging,” leading to increased susceptibility to infections and conditions like periodontitis (39). The impaired mTOR signaling pathway in diabetes further contributes to immune suppression by inhibiting the respiratory burst necessary for effective pathogen clearance, highlighting a mechanistic link between metabolic dysregulation and immune dysfunction (40).

These processes exemplify how basal inflammation, often exacerbated by chronic hyperglycemia, can significantly impair the immune system’s ability to respond to infections and vaccinations through trained immunity, making diabetic individuals more susceptible to both infections and poor vaccine responses. **Table 1** summarizes the common causes of impaired mucosal immunity in diabetics.

**Table 1:** Causes of Impaired Mucosal Immunity in Diabetes.

Cause	Description	References
Gut Microbiota Dysbiosis	Diabetes disrupts gut microbiota balance, reducing beneficial metabolites and weakening mucosal barrier integrity and promoting systemic inflammation.	(41–43)
Increased Intestinal Permeability	Hyperglycemia damages tight intestinal junctions ("leaky gut"), allowing bacterial toxins (e.g., LPS) into circulation, triggering chronic inflammation.	(41, 43, 44)
Impaired IL-22 Signaling	Reduced IL-22 levels (critical for mucosal repair) due to defects in IL-23/IL-22 axis activation, weakening antimicrobial defenses.	(41, 43, 44)
Chronic Low-Grade Inflammation	Persistent inflammation from adipose-derived cytokines (TNF-α, IL-6) disrupts mucosal immune homeostasis and suppresses protective responses.	(39, 41, 45)
Hyperglycemia-Induced Oxidative Stress	High glucose generates ROS and AGEs, damaging mucosal epithelial/immune cells and impairing immune function.	(39, 41, 45)
Altered Immune Cell Populations	Dysfunctional innate lymphoid cells (ILCs) and unconventional T cells reduce barrier defense and gut-pancreas communication.	(42, 44, 45)
Disrupted Gut-Pancreas Axis	Autoimmune cross-reactivity in type 1 diabetes links gut mucosal immunity to pancreatic β-cell destruction, exacerbated by environmental triggers (e.g., viruses).	(41–43)
Reduced Antimicrobial Peptides	Decreased defensins and other antimicrobial peptides at mucosal sites increase susceptibility to pathogens (e.g., *Citrobacter rodentium*).	(43, 44, 46)
Endoplasmic Reticulum (ER) Stress	Hyperglycemia-induced ER stress impairs intestinal epithelial regeneration and barrier maintenance, disrupting immune signaling.	(41, 44, 45)
Immune Cell Metabolic Dysregulation	Altered glycolysis and lipid metabolism in immune cells reduce their capacity to respond effectively at mucosal surfaces.	(41, 44, 45)

## Influence of glycemic control on vaccine effectiveness

**Figure 1:**
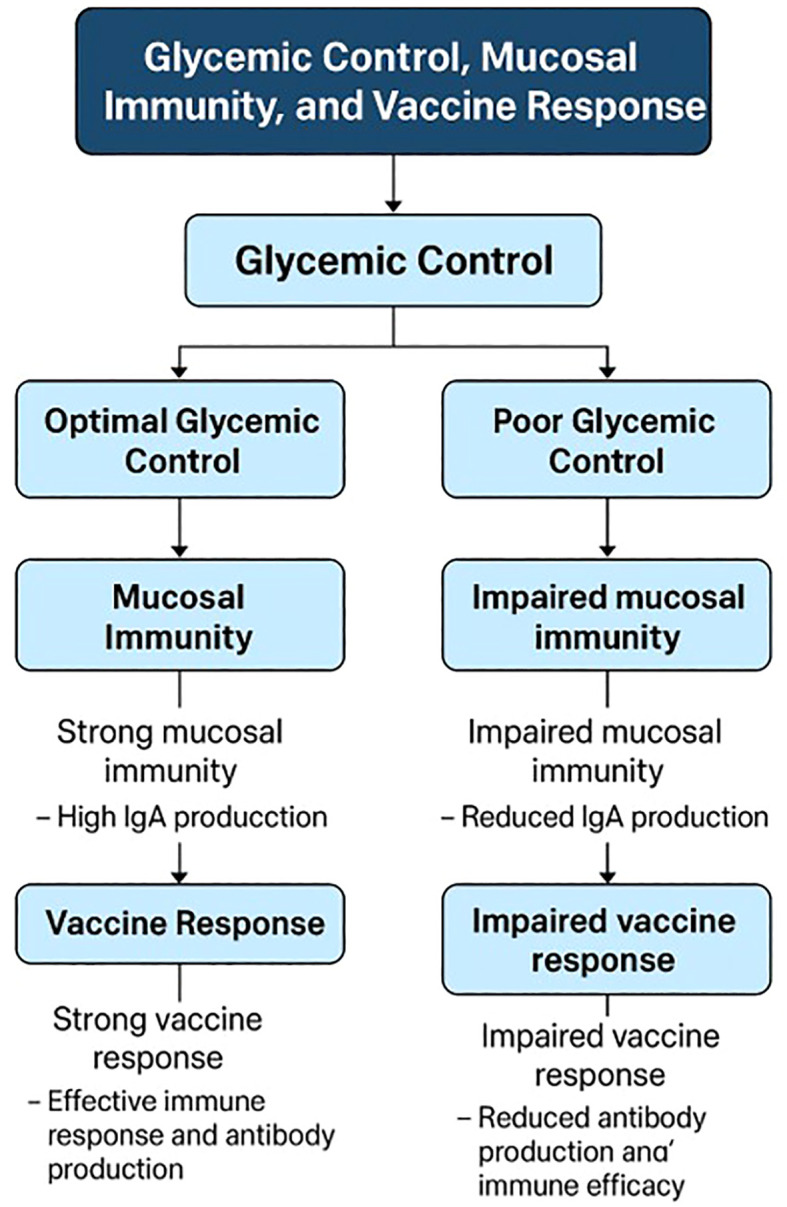
Interplay between glycemic control, mucosal immunity, and vaccine response.

Glycemic control significantly influences the effectiveness of vaccines in patients with diabetes, as evidenced by various studies focusing on different vaccines. For instance, in the context of COVID-19 vaccines, poor glycemic control in patients with type 2 diabetes is associated with lower immune responses and a higher incidence of breakthrough infections following mRNA-BNT162b2 vaccination. Patients with better glycemic control (HbA1c < 7%) demonstrated higher virus-neutralizing antibody capacity and a better CD4+ T/cytokine response compared to those with poor control (HbA1c ≥ 7%) (18). Similarly, a systematic review highlighted that patients with diabetes vaccinated with inactivated COVID-19 vaccines like CoronaVac/SinoVac and BBV-152 had lower seroconversion rates compared to non-diabetic individuals, emphasizing the importance of glycemic management to enhance vaccine immunogenicity (12). In older adults with diabetes, altered glycemic control can stimulate proinflammatory mediators, increasing infection risk, although vaccines like the influenza vaccine have been shown to reduce hospitalization and mortality rates without affecting glycemic control (47). Furthermore, a study in Sri Lanka found that while glycemic control did not significantly affect seroconversion rates post-Sinopharm COVID-19 vaccination, a third of diabetic patients did not achieve protective antibody levels, indicating the need for improved glycemic management (48). In type 1 diabetes, pre-vaccination glucose control, particularly maintaining glucose time in range (TIR), was associated with stronger antibody responses to the SARS-CoV-2 vaccine, underscoring the role of well-controlled blood glucose in enhancing vaccine efficacy (13). A key value here is the optimization of immune function through metabolic control, ensuring that diabetic patients can mount an effective response to vaccination (13). These findings collectively suggest that maintaining optimal glycemic control is crucial for maximizing the effectiveness of vaccines, including those for COVID-19, influenza, and other infectious diseases, in diabetic populations. **Figure 1** provides a schematic diagram illustrating the complex interplay between glycemic control, mucosal immunity, and vaccine response.

## Enhancing vaccine response in diabetics

Enhancing vaccine response in diabetics, particularly through improved glycemic control, is a multifaceted challenge that involves understanding the interplay between diabetes management and immune function. Poor glycemic control in diabetic patients has been associated with a diminished immune response to vaccines, including COVID-19 vaccines, as evidenced by lower antibody levels compared to non-diabetic individuals (10–12). This reduced immunogenicity is exacerbated by factors such as higher age and BMI, which are common in diabetic populations (10). To address this, continuous glucose monitoring (CGM) has been suggested as a cost-effective strategy to optimize glycemic control around the time of vaccination, potentially enhancing the immune response (11). Moreover, transient increases in blood glucose levels post-vaccination have been observed, indicating the need for careful monitoring and management of insulin resistance during this period (14). The importance of maintaining optimal glycemic control is further underscored by findings that diabetic patients with well-managed blood sugar levels exhibit immune responses comparable to healthy controls (49). Additionally, innovative approaches such as the use of oral vaccines, as demonstrated in animal models, show promise in reversing diabetes and improving immune responses by reducing islet inflammation and promoting tolerance (50). While these strategies primarily focus on systemic immunity, enhancing mucosal immunity could also be beneficial. Techniques like Transient Microbiota Depletion-boosted Immunization (TMDI) have been shown to enhance mucosal immune responses by expanding tissue-resident memory T cells, suggesting a potential avenue for improving mucosal immunity in diabetics (51). An unresolved issue is the long-term durability of vaccine-induced immunity in diabetic patients, particularly given the potential for waning antibody levels and the need for booster doses (12, 52). Overall, integrating glycemic control with novel immunization strategies could significantly enhance vaccine efficacy in diabetic patients, although further research is needed to refine these approaches and address unresolved issues such as vaccine type and administration frequency.

Adjunct therapies play a significant role in enhancing vaccine efficacy by modulating the immune response through various mechanisms. Probiotics, for instance, have emerged as promising adjuvants due to their ability to enhance both mucosal and systemic immunity, stimulate cytokine production, and regulate T-cell activity. Specific strains like *Lactobacillus rhamnosus* GG have been shown to improve immune responses to vaccines against rotavirus, SARS-CoV-2, and influenza, and they also hold potential in cancer immunotherapy by promoting T cell infiltration and inhibiting tumor growth (53). Traditional adjuvants, such as alum, enhance vaccine efficacy by protecting antigens from rapid degradation and directing immune responses towards either cell-mediated or antibody production pathways (54). Physical adjuvants, like radiofrequency treatment, offer a non-invasive alternative that can safely boost humoral and cellular immune responses, as demonstrated in murine models for H1N1 influenza vaccination (55). Additionally, micronutrient supplementation, particularly with vitamins and minerals, has been shown to improve vaccine efficacy by supporting lymphocyte function and enhancing antibody titers, which is crucial for populations with deficiencies (56). A significant value in using micronutrient supplementation is addressing underlying deficiencies that can impair immune function, thereby improving the overall health and responsiveness of the individual to vaccination (57). In pediatric populations, optimizing vaccine efficacy involves using adjuvants and personalized vaccination schedules to accommodate the developmental state of children’s immune systems, thereby reducing post-vaccination complications (58). Furthermore, complementary and alternative medicine approaches, such as the use of resveratrol, green tea, and curcumin, have shown potential in modulating immune responses and could be integrated into vaccine strategies to enhance efficacy (59). Overall, these adjunct therapies, whether through biological, chemical, or physical means, provide diverse and promising avenues to boost vaccine efficacy across different populations and disease contexts.

Dietary and lifestyle interventions play a crucial role in enhancing mucosal immune responses in individuals with diabetes, primarily through the modulation of gut microbiota and the incorporation of specific nutrients. The Mediterranean diet, rich in omega-3 fatty acids, probiotics, and prebiotics, has been shown to improve immune function and manage diabetes by exerting anti-inflammatory effects (60). The gut microbiota, a key player in immune modulation, can be influenced by dietary interventions such as the inclusion of short-chain fatty acids (SCFAs), which have been shown to reshape the gut environment and reduce systemic inflammation associated with diabetes (61). Furthermore, a gluten-free, hydrolyzed casein diet has been demonstrated to normalize inflammatory markers and β-cell chemokine expression in diabetic models, highlighting the impact of diet on immune responses (62). Nutrients such as vitamins, micronutrients, and bioactive compounds like coenzyme Q10 and alpha-lipoic acid are essential for maintaining immune homeostasis and have been linked to improved immune function in type 2 diabetes (63). The gut-associated lymphoid tissue, a critical site for mucosal immunity, is significantly influenced by nutritional elements, which affect lymphocyte activity and cytokine production, thereby enhancing the body’s defense mechanisms (64). Additionally, the strategic manipulation of the gut microbiota through dietary practices can improve glycemic control and reduce diabetes-related complications by influencing insulin sensitivity and inflammation (65, 66). Physical activity, stress management, and adequate sleep are also vital lifestyle modifications that support immune function and glycemic control in diabetics (60). A key issue is the adherence to long-term dietary and lifestyle changes, which can be challenging for many individuals, requiring ongoing support and education (67, 68). Overall, a comprehensive approach that combines dietary modifications with lifestyle changes can significantly enhance mucosal immune responses and improve diabetes management.

## Future directions and recommendations

Future research should focus on developing personalized vaccination strategies for individuals with diabetes, given the unique challenges posed by this condition. One promising approach could be the development of mucosal vaccines, which are specifically designed to target the entrance points of pathogens, such as the respiratory and gastrointestinal tracts. Mucosal vaccines offer the advantage of inducing local immune responses, including the production of secretory IgA, which are crucial for preventing infections at these sites. This is particularly important for diabetic patients, who may have impaired systemic immunity but can still benefit from localized protection.

Vaccination schedules—including the timing of initial doses and subsequent booster shots—could be tailored according to key patient-specific factors such as glycemic control, age, body mass index (BMI), and kidney function, all of which can influence the effectiveness of the immune response in diabetic individuals. For example, patients with poor glycemic control may benefit from adjusted dosing schedules or additional booster doses to overcome the dampened immune response often observed in hyperglycemic states. On the other hand, those with better glycemic control could potentially benefit from standard vaccination regimens, as their immune responses may more closely resemble those of non-diabetic individuals. In addition, taking into account factors such as age and BMI, which are known to influence immunogenicity, should be considered to optimize vaccine formulations and administration schedules for different subgroups within the diabetic population. In individuals with compromised kidney function, an important factor given the prevalence of diabetic nephropathy, personalized schedules may also help improve vaccine-induced protection by mitigating the impact of renal impairment on immune responses.

There is also potential for mucosal vaccines to play a particularly valuable role in diabetic populations. These vaccines may provide more effective localized immunity, reducing the risk of infection at common pathogen entry points. Further studies are needed to explore the efficacy and safety of mucosal vaccines in diabetic patients, which could lead to more effective and patient-specific vaccination strategies.

In parallel with personalized approaches, future clinical trials should incorporate comprehensive endpoints that assess both systemic and mucosal immunity. Traditionally, vaccine studies have focused primarily on serum antibody titers; however, the evaluation of mucosal immunity—particularly secretory IgA responses at the entry points of pathogens—can provide a more complete picture of vaccine efficacy. Designing trials that capture a dual analysis of systemic and local immune responses will be critical for understanding the full spectrum of protective immunity, especially in populations where mucosal defenses may be compromised. Moreover, exploring novel mucosal vaccine candidates and innovative adjuvant combinations in these trials could lead to formulations that are better suited for inducing robust and durable mucosal immunity, thereby enhancing overall protection against infectious diseases.

Finally, an interdisciplinary approach is essential to maximize vaccine efficacy in patients with diabetes. Integrating nutritional, metabolic, and immunological interventions can create a synergistic effect that improves vaccine responses. For instance, addressing nutritional deficiencies—such as *that of* vitamin A, which is vital for mucosal immune function—could help restore a balanced immune environment and support more effective vaccine responses. Similarly, optimizing metabolic control through dietary adjustments, physical activity, and medical management could reduce chronic inflammation and improve immune cell function. When these strategies are combined with advanced immunological insights, they hold the promise of establishing a holistic framework that not only enhances vaccine-induced immunity but also improves the overall health and resilience of individuals with diabetes.

## Conclusion

In conclusion, the available evidence underscores that glycemic control is a critical determinant of vaccine responsiveness in diabetic patients, with significant implications for both systemic and mucosal immunity. Poorly controlled blood glucose levels are associated with diminished antibody responses post-vaccination, whereas optimal glycemic management appears to enhance immunogenicity, particularly by bolstering mucosal defenses such as secretory IgA production. These mucosal responses are crucial for preventing pathogen entry at their primary sites, thereby providing an essential first line of protection in diabetic individuals who are at heightened risk of severe infections. Moreover, evidence indicates that a personalized approach to vaccination—tailoring booster schedules and vaccine regimens based on factors such as glycemic status, age, BMI, and kidney function—may be necessary to overcome the immunological challenges associated with diabetes. By integrating continuous glucose monitoring and other metabolic assessments into vaccination strategies, clinicians can better predict and enhance vaccine-induced immunity in this vulnerable population. Moreover, the data suggest that a personalized approach to vaccination tailoring booster schedules and vaccine regimens based on factors like glycemic status, age, BMI, and kidney function may be necessary to overcome the immunological challenges associated with diabetes. By integrating continuous glucose monitoring and other metabolic assessments into vaccination strategies, clinicians can better predict and enhance vaccine-induced immunity in this vulnerable population. Additionally, adjunct therapies, including nutritional and probiotic interventions, hold promise for further modulating the gut microbiota and reinforcing mucosal barrier integrity. Such strategies may not only improve glycemic control but also augment the mucosal immune response, thereby optimizing overall vaccine efficacy. A critical value is the holistic approach to patient care, integrating multiple interventions to address the complex interplay of factors affecting vaccine response in diabetic patients. Ultimately, an interdisciplinary approach that combines metabolic management with innovative vaccine design and personalized immunization schedules is essential to maximize protection in diabetic patients. Future longitudinal studies are needed to refine these strategies and to determine the optimal timing and formulation of booster doses that can sustain robust immune protection over time.

## References

[1] Akdeniz YS Piskinpasa H Esen A Polat Ö Tevetoglu IO Ogredici G. The glycemic control difference in type 1 and type 2 diabetic patients. Global J Endocrine Metab. (2018) 2. doi: 10.31031/GJEM.2018.02.000528

[2] Kamanzi NG. Various diabetes management activities: A comprehensive overview. IDOSR J Biochem Biotechnol Allied Fields. (2024) 9:24–36. doi: 10.59298/idosr/jbbaf/24/93.3540000

[3] Mohammed AS Ouda WE-S Ali E. A. E.-F. Study of glycemic control and management of children suffering from type 1 diabetes versus type 2 diabetes. Egyptian J Health Care. (2024) 15:436–47. doi: 10.21608/ejhc.2024.339280

[4] Cheng LJ Wang W Lim ST Wu VX. Factors associated with glycaemic control in patients with diabetes mellitus: A systematic literature review. J Clin Nurs. (2019) 28:2090–111. doi: 10.1111/JOCN.14795 30667583

[5] Hu Z Fu Y-X Peng H. Mucosal vaccine development for respiratory viral infections. Health Life. (2023) 1:281–8. doi: 10.1016/j.hlife.2023.12.005

[6] Park S Wiest MJ Yan V Wong PT Schotsaert M. Induction of protective immune responses at respiratory mucosal sites. Hum Vaccines Immunotherapeutics. (2024) 20:2368288. doi: 10.1080/21645515.2024.2368288 PMC11221474 38953250

[7] Li X. Exploring mucosal vaccination as an alternative to conventional vaccines. Proc SPIE. (2024) 12988:129881I. doi: 10.1117/12.3012823

[8] Song Y Mehl F Zeichner SL. Vaccine strategies to elicit mucosal immunity. Vaccines. (2024) 12:191. doi: 10.3390/vaccines12020191 38400174 PMC10892965

[9] Tsai CJ Fujihashi K. Mucosal vaccine delivery. In: Vaccine Delivery Technology. Amsterdam, Netherlands: Elsevier (2024). p. 89–107. doi: 10.1016/b978-0-443-18564-9.00005-9

[10] Boroumand AB Forouhi M Karimi F Moghadam AS Naeini LG Kokabian P. Immunogenicity of COVID-19 vaccines in patients with diabetes mellitus: A systematic review. Front Immunol. (2022) 13:940357. doi: 10.3389/fimmu.2022.940357 36105809 PMC9465310

[11] Pieralice S D’Onofrio L Pozzilli P Buzzetti R. Third dose of COVID-19 vaccine in diabetes: Relevance of good metabolic control to improve its efficacy. Diabetes/Metabolism Res Rev. (2022) 38:e3533. doi: 10.1002/dmrr.3533 PMC9087402 35468252

[12] Prasetyaningtyas D Soegiarto G Wulandari L. Effects of diabetes mellitus regulation on antibody response to inactivated virus vaccine: a systematic review. Bali Med J. (2023) 12:1575–80. doi: 10.15562/bmj.v12i2.4171

[13] Alhamar GE Briganti S Maggi D Viola V Faraj M Zannella C. Pre-vaccination glucose time in range correlates with antibody response to SARS-CoV-2 vaccine in type 1 diabetes. J Clin Endocrinol Metab. (2023) 108:1673–82. doi: 10.1210/clinem/dgad001 PMC10807908 36611249

[14] Zilbermint M Motevalli M Batty K Venner-Walcott J Edwards A Burley T. Effects of the COVID-19 booster vaccine on glycemia and insulin resistance in people with type 1 diabetes: A prospective pilot study. Diabetes Res Clin Pract. (2023) 203:110898. doi: 10.1016/j.diabres.2023.110898 37678726

[15] Shomali N Mahmoudi J Mahmoodpoor A Zamiri RE Akbari M Xu H. Harmful effects of high amounts of glucose on the immune system: An updated review. Biotechnol Appl Biochem. (2021) 68:484–90. doi: 10.1002/BAB.1938 32395846

[16] Kolay SR Sinha RP Dubey NK. Diabetes and the immune system: Understanding the intricate relationship. Res Reviews: J Dairy Sci Technol. (2024) 2:28. doi: 10.58532/nbennurrdch28

[17] Ye Z Li L Yang L Li Z Aspatwar A Wang L. Impact of diabetes mellitus on tuberculosis prevention, diagnosis, and treatment from an immunologic perspective. Exploration. (2024) 4:20230138. doi: 10.1002/exp.20230138 39439490 PMC11491313

[18] Marfella R Sardu C D’Onofrio N Prattichizzo F Scisciola L Messina V. Glycaemic control is associated with SARS-CoV-2 breakthrough infections in vaccinated patients with type 2 diabetes. Nat Commun. (2022) 13:2428. doi: 10.1038/s41467-022-30068-2 35484164 PMC9051134

[19] MacIver NJ Jacobs SR Wieman HL Wofford JA Coloff JL Rathmell JC. Glucose metabolism in lymphocytes is a regulated process with significant effects on immune cell function and survival. J Leukocyte Biol. (2008) 84:949–57. doi: 10.1189/JLB.0108024 PMC2638731 18577716

[20] Olayanju OA Mba IN Akinmola OO Awah NE Ofagbor E Okonkwo O. Original: Relationship between glycaemic control and oral immunologic proteins. West Afr J Med. (2022) 39(10):1062–7. 36260823

[21] Moin ASM Sathyapalan T Atkin SL Butler AE. 393-P: Acute hypoglycemia alters serum immune mediators associated with macrophage and T-cell activation in type 2 diabetes. Diabetes. (2023) 72:393–P. doi: 10.2337/db23-393-p

[22] Sinha D Yaugel-Novoa M Waeckel L Paul S Longet S. Unmasking the potential of secretory IgA and its pivotal role in protection from respiratory viruses. Antiviral Res. (2024) 223:105823. doi: 10.1016/j.antiviral.2024.105823 38331200

[23] Takahashi I Nochi T Kunisawa J Yuki Y Kiyono H. The mucosal immune system for secretory IgA responses and mucosal vaccine development. Inflammation Regeneration. (2010) 30:40–8. doi: 10.2492/INFLAMMREGEN.30.40

[24] Fujihashi K Boyaka PN McGhee JR. The mucosal immune response. In: Topley and Wilson’s Microbiology and Microbial Infections, 10th ed, vol. 1. New Jersey, USA: John Wiley & Sons, Ltd (2010). p. 227–58. doi: 10.1002/9780470688618.TAW0109

[25] Corrêa V Portilho AI Gaspari ED. Vaccines, adjuvants and key factors for mucosal immune response. Immunology. (2022) 167:16–29. doi: 10.1111/imm.13526 35751397

[26] Monjori M. Mucosal immunology and vaccination. J Pediatr Neurosci. (2010) 5:45. doi: 10.4103/1817-1745.66676 21042509 PMC2964793

[27] Kunisawa J Gohda M Kiyono H. Uniqueness of the mucosal immune system for the development of prospective mucosal vaccine. ChemInform. (2007) 38:319–26. doi: 10.1002/CHIN.200721274 17268152

[28] Anjuère F Czerkinsky C. Immunité muqueuse et vaccination [Mucosal immunity and vaccination. M S-Medecine Sci. (2007) 23:371–6. doi: 10.1051/MEDSCI/2007234371 17433226

[29] Holmgren J Czerkinsky C. Mucosal immunity and vaccines. Nat Med. (2005) 11:S45–53. doi: 10.1038/NM1213 15812489

[30] Thaiss CA Levy M Grosheva I Zheng D Soffer E Blacher E. Hyperglycemia drives intestinal barrier dysfunction and risk for enteric infection. Science. (2018) 359:1376–83. doi: 10.1126/SCIENCE.AAR3318 29519916

[31] Chu J Feng S Guo C Xue B He K Li L. Immunological mechanisms of inflammatory diseases caused by gut microbiota dysbiosis: A review. Biomedicine Pharmacotherapy. (2023) 164:114985. doi: 10.1016/j.biopha.2023.114985 37311282

[32] Wang L Lin W Lei K Wang H Zhang X Jiang S. Hyperglycemia-enhanced neutrophil extracellular traps drive mucosal immunopathology at the oral barrier. Advanced Sci. (2024) 11:e202407346. doi: 10.1002/advs.202407346 PMC11653653 39499780

[33] Dubois N Muñoz-García J Heymann D Renodon-Cornière A. High glucose exposure drives intestinal barrier dysfunction by altering its morphological, structural and functional properties. Biochem Pharmacol. (2023) 216:115765. doi: 10.1016/j.bcp.2023.115765 37619641

[34] Dutton JS Hinman SS Kim R Attayek PJ Maurer M Sims CS. Hyperglycemia minimally alters primary self-renewing human colonic epithelial cells while TNFα-promotes severe intestinal epithelial dysfunction. Integr Biol. (2021) 13:87–101. doi: 10.1093/INTBIO/ZYAB008 PMC8204630 33989405

[35] Minton K. Mucosal immunology: Glucose not good for the gut. Nat Rev Immunol. (2018) 18:223. doi: 10.1038/NRI.2018.22 29561547

[36] Rouland M Beaudoin L Rouxel O Bertrand L Cagninacci L Saffarian A. Gut mucosa alterations and loss of segmented filamentous bacteria in type 1 diabetes are associated with inflammation rather than hyperglycaemia. Gut. (2021) 70:2055–66. doi: 10.1136/GUTJNL-2020-323664 33593807

[37] Markelova E Golitsyna AA Yugai YV Pervov YY Kovalchuk VK. Features of mucosal immunity in the development of periodontal diseases in patients with type II diabetes mellitus. Russian J Immunol. (2022) 25:439–46. doi: 10.46235/1028-7221-1165-fom

[38] Sorini C Cosorich I Conte ML Giorgi LD Facciotti F Lucianò R. Loss of gut barrier integrity triggers activation of islet-reactive T cells and autoimmune diabetes. Proc Natl Acad Sci United States America. (2019) 116:15140–9. doi: 10.1073/PNAS.1814558116 PMC6660755 31182588

[39] Alexander MP Cho E Gliozheni E Salem YM Cheung JKH Ichii H. Pathology of diabetes-induced immune dysfunction. Int J Mol Sci. (2024) 25:7105. doi: 10.3390/ijms25137105 39000211 PMC11241249

[40] Genito CJ Darwitz B Thurlow LR. Impaired mTOR signaling causes immune suppression in diabetes. J Immunol. (2023) 210:160.04. doi: 10.4049/jimmunol.210.supp.160.04

[41] Daryabor G Atashzar MR Kabelitz D Meri S Kalantar K. The effects of type 2 diabetes mellitus on organ metabolism and the immune system. Front Immunol. (2020) 11:1582. doi: 10.3389/fimmu.2020.01582 32793223 PMC7387426

[42] Liu R Zhang J Chen S Xiao Y Hu J Zhou Z. Intestinal mucosal immunity and type 1 diabetes: Non-negligible communication between gut and pancreas. Diabetes Obesity Metab. (2025) 27:1045–64. doi: 10.1111/dom.16101 PMC11802406 39618164

[43] Wang X Ota N Manzanillo P Kates L Zavala-Solorio J Eidenschenk C. Interleukin-22 alleviates metabolic disorders and restores mucosal immunity in diabetes. Nature. (2014) 514:237–41. doi: 10.1038/nature13564 25119041

[44] Zhou X Wu Y Zhu Z Lu C Zhang C Zeng L. Mucosal immune response in biology, disease prevention and treatment. Signal Transduction Targeted Ther. (2025) 10:7. doi: 10.1038/s41392-024-02043-4 PMC11707400 39774607

[45] Donath MY Dinarello CA Mandrup-Poulsen T. Targeting innate immune mediators in type 1 and type 2 diabetes. Nat Rev Immunol. (2019) 19:734–46. doi: 10.1038/s41577-019-0213-9 31501536

[46] Akash MSH Rehman K Fiayyaz F Sabir S Khurshid M. Diabetes-associated infections: Development of antimicrobial resistance and possible treatment strategies. Arch Microbiol. (2020) 202:953–65. doi: 10.1007/s00203-020-01818-x PMC7223138 32016521

[47] Almasri L Holtzclaw BJ. Assessing vaccine protection for older adults with diabetes: A systematic review. Western J Nurs Res. (2021) 43:788–99. doi: 10.1177/01939459211005710 33845695

[48] Kottahachchi DC Badanasinghe N Samarathunga P Sandeepani P Cooray S Warnakulasuriya T. The effect of glycaemic control on neutralizing antibody response to COVID-19 among patients with Type 2 diabetes mellitus in the Kurunegala District of Sri Lanka; A prospective cohort study. Sri Lanka J Diabetes Endocrinol Metab. (2023) 14:84–93. doi: 10.4038/sjdem.v14i2.7510

[49] Vasilev G Kabakchieva P Miteva D Batselova HM Velikova T. Effectiveness and safety of COVID-19 vaccines in patients with diabetes as a factor for vaccine hesitancy. World J Diabetes. (2022) 13:738–52. doi: 10.4239/wjd.v13.i9.738 PMC9521442 36188150

[50] Cobb J Rawson J Gonzalez N Orr C Kandeel F Husseiny MI. Reversal of diabetes by an oral Salmonella-based vaccine in acute and progressive diabetes in NOD mice. PloS One. (2024) 19:e0303863. doi: 10.1371/journal.pone.0303863 38781241 PMC11115281

[51] Becattini S Littmann ER Seok R Amoretti LA Fontana E Wright RJ. Enhancing mucosal immunity by transient microbiota depletion. Nat Commun. (2020) 11:4499. doi: 10.1038/S41467-020-18248-4 32901029 PMC7479140

[52] Pal R Bhadada SK Misra A. COVID-19 vaccination in patients with diabetes mellitus: Current concepts, uncertainties and challenges. Diabetes Metab Syndrome: Clin Res Rev. (2021) 15:505–8. doi: 10.1016/J.DSX.2021.02.026 PMC7904463 33662837

[53] Abavisani M Ebadpour N Khoshrou A Sahebkar A. Boosting vaccine effectiveness: The groundbreaking role of probiotics. J Agric Food Res. (2024) 16:101189. doi: 10.1016/j.jafr.2024.101189

[54] Chodaczek G. Adjuvants as factors improving efficiency of vaccination. Postepy Higieny i Medycyny Doswiadczalnej (Online). (2004) 58:47–59. 15069382

[55] Cao Y Zhu X Hossen N Kakar P Zhao Y Chen X. Augmentation of vaccine-induced humoral and cellular immunity by a physical radiofrequency adjuvant. Nat Commun. (2018) 9:3654. doi: 10.1038/S41467-018-06151-Y 30209303 PMC6135850

[56] Torshin IY Gromova OA Maksimov VA Chuchalin AG. Improving the effectiveness of vaccination against viral and bacterial pathogens through micronutrient supplementation. Pul′monologiâ. (2022) 32:230–41. doi: 10.18093/0869-0189-2022-2356

[57] Peace CG O’Neill LAJ. Ironing out vaccine efficacy. Med. (2021) 2:116–8. doi: 10.1016/J.MEDJ.2021.01.003 PMC8837395 35187513

[58] Zuxra A. Methods of increasing the effectiveness of vaccines in children and reducing post-vaccination complications. Int J Med Sci Clin Res. (2024) 4:24–9. doi: 10.37547/ijmscr/volume04issue09-06

[59] Clarke JO Mullin GE. A review of complementary and alternative approaches to immunomodulation. Nutr Clin Pract. (2008) 23:49–62. doi: 10.1177/011542650802300149 18203964

[60] Srividya N Haldipur AC Yerra H. Lifestyle modifications and nutritional modulation of immune system for prevention and management of diabetes mellitus: Current perspectives. In: Modulation of Human Immunity through Plant-Based Interventions. Amsterdam, Netherlands: Elsevier (2024). p. 325–46. doi: 10.1016/b978-0-443-13195-0.00016-8

[61] Yap YA Mariño E. Dietary SCFAs immunotherapy: Reshaping the gut microbiota in diabetes. In: Short-Chain Fatty Acids: Metabolism, Mechanisms of Action and Implications in Human Health. Springer, Cham (2020). p. 203–22. doi: 10.1007/5584_2020_515

[62] Henschel AM Cabrera SM Kaldunski ML Jia S Geoffrey R Roethle MF. Modulation of the diet and gastrointestinal microbiota normalizes systemic inflammation and β-cell chemokine expression associated with autoimmune diabetes susceptibility. PloS One. (2018) 13:e0190351. doi: 10.1371/JOURNAL.PONE.0190351 29293587 PMC5749787

[63] Napiórkowska-Baran K Treichel P Czarnowska M Drozd M Koperska K Węglarz A. Immunomodulation through nutrition should be a key trend in type 2 diabetes treatment. Int J Mol Sci. (2024) 25:3769. doi: 10.3390/ijms25073769 38612580 PMC11011461

[64] Cunningham-Rundles S. Nutrition and the mucosal immune system. Curr Opin Gastroenterology. (2001) 17:185–9. doi: 10.1097/00001574-200103000-00013 11224675

[65] Hamamah S Iatcu OC Covașă M. Nutrition at the intersection between gut microbiota eubiosis and effective management of type 2 diabetes. Nutrients. (2023) 16(2):269. doi: 10.20944/preprints202312.1651.v1 PMC10820857 38257161

[66] Patel N Dinesh S Sharma S. From gut to glucose: A comprehensive review on functional foods and dietary interventions for diabetes management. Curr Diabetes Rev. (2023) 20:e270923221534. doi: 10.2174/0115733998266653231005072450 37861021

[67] Franz MJ. Nutrition therapy for the prevention and treatment of prediabetes and diabetes. In: Evert LM Evert AB, editors. Nutrition Therapy for Diabetes. Berlin, Germany: Springer International Publishing (2017). p. 141–68. doi: 10.1007/978-3-319-43027-0_8

[68] Siracusa F Tintelnot J Cortesi F Gagliani N. Diet and immune response: how today’s plate shapes tomorrow’s health. Trends Immunol. (2023) 44:938–51. doi: 10.1016/j.it.2023.10.010 37949784

